# Supplementary material for: Deletion of wfs1 Impairs Oligodendrocyte Precursor Cells Dorsal Distribution and Myelination Through the wfs1-hmgcs1 Axis in Zebrafish
Source: Biology (Basel). 2026 Aug 16;15(16):1402. doi: 10.3390/biology15161402 (PMC13510151; doi:10.3390/biology15161402)
Supplement: Supplementary file 1 [file biology-15-01402-s001.zip › File S1-Transcriptome sequencing data and differentially expressed genes (DEGs) in wfs1-- zebrafish at 5 dpf/2.KEGG/wfs1KOvsWT/UP/src/dre04912.html]

dre04912


- dre:100006675

- Up regulated genes

100006675(1.63301233543285)

- dre:568726

- Up regulated genes

568726(1.14419230813088)
- dre:560807

- Up regulated genes

560807(1.71247301071773)

- dre:793158

- Up regulated genes

793158\_1(1.86160461223548)
- dre:556226

- Up regulated genes

556226\_1(6.36622858283894)

- dre:556410

- Up regulated genes

556410\_1(1.7264669285964)

- dre:562552

- Up regulated genes

562552(1.50682290794657)

- dre:192322

- Up regulated genes

336121(1.42904963424916)

- dre:565743

- Up regulated genes

565743\_1(2.40489825782141)

- dre:30498

- Up regulated genes

30498\_1(7.44988431851508)

Close
